# The optimized inclusion level of *Bacillus subtilis* fermented *Azolla pinnata* in Nile tilapia (*Oreochromis niloticus)* diets: immunity, antioxidative status, intestinal digestive enzymes and histomorphometry, and disease resistance

**DOI:** 10.1007/s10695-022-01076-2

**Published:** 2022-04-30

**Authors:** Taha Ismail, Elsayed Hegazi, Eldsokey Nassef, Ola A. Habotta, Mahmoud S. Gewaily

**Affiliations:** 1 Department of Nutrition and Clinical Nutrition, Faculty of Veterinary Medicine, Kafrelsheikh University, Kafrelsheikh, 33516 Egypt; 2 Department of Forensic Medicine and Toxicology, Faculty of Veterinary Medicine, Mansoura University, Mansoura, 35516 Egypt; 3 Department of Anatomy and Embryology, Faculty of Veterinary Medicine, Kafrelsheikh University, Kafrelsheikh, 33516 Egypt

**Keywords:** *Azolla pinnata*, Nile tilapia, Intestinal digestive enzymes, Solid state fermentation, Antioxidative status

## Abstract

A 95-day feeding trial was conducted to evaluate the outcomes of feeding *Bacillus subtilis* fermented *Azolla* (*BSFA*) on nonspecific immunity, antioxidative status, intestinal digestive enzymes and histomorphometry, and disease resistance in the Nile tilapia. We formulated five isonitrogenous and isocaloric experimental diets to incorporate *BSFA* at level of (0%, 15%, 30%, 45%, 60%). The growth performance parameters (FBW, BWG, SGR, PER, and FCR) revealed a significant increase in the BSFA30 tilapia group compared to the control group followed by *BSFA45* (*P* < 0.05). The *BSFA30* group exhibited the highest nonspecific immunity parameters including (lysozyme activity, phagocytic index, and phagocytic activity) compared to other groups (*P* < 0.05). SOD and GPx reported the highest values in the *BSFA60* group. Nile tilapia carcass composition was not influenced by *BSFA* inclusion level (*P* > 0.05). Interestingly, Nile tilapia fed with *BSFA15* diet exhibited the highest protease activity level (*P* < 0.05), while those fed on *BSFA30* documented the highest amylase activity. Intestinal histomorphology was significantly enhanced with the gradual increase of administrated *BSFA*. Regarding the tilapia disease resistance against *Aeromonas septicemia*, *BSFA* significantly diminished the cumulative mortality compared to the control group. To sum up, *BSFA* was more effective in improving the growth performance and immunity of Nile tilapia.

**Graphical abstract:**

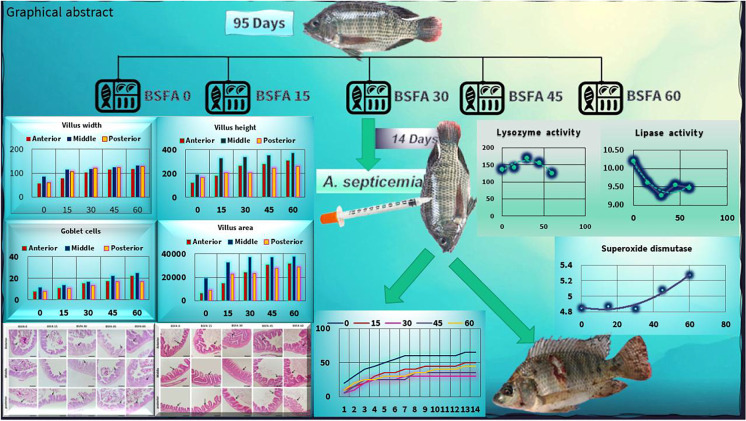

## Introduction

*Azolla*, a small fast-growing aquatic plant, has several environmental drawbacks. It was reported for its effect on biological diversity through threatening local fauna and rare species communities (Sax et al. 2005; Vander Zanden and Olden 2008). Also, it negatively affects the aquatic ecosystem due to its role in diminishing oxygen and nutrient contents and enhancing water turbidity (Olenin et al. 2007).

From the nutritional perspective, *Azolla pinnata* is characterized by its higher availability and nutritive value (Vahedi et al. 2021). It grows in association with a nitrogen fixing organism called *Anabaena azollae* (Prabina and Kumar 2010). *A. pinnata* contains higher protein content with a characteristic essential amino acid profile than other green forage crops and most macrophytes (Cherryl et al. 2014). Interestingly, at different inclusion levels, it improves growth performance and feed utilization in *Labeo rohita* (Datta 2011), *Oreochromis niloticus* (Magouz et al. 2020), *Gervais* (Abdel-Tawwab 2008), *Cyprinus carpio* (Abdel-Tawwab 2006). Despite its nutritional value, *Azolla* inclusion levels in tilapia diets are extremely limited in aquaculture due to its low protein digestibility and high fiber content (Leonard et al. 1998).

Nile tilapia is one of the most important fast-growing fish in the world, with an annual production of 4525.4 tons in 2018; thus, it represents about 8.3% of the global aquaculture market share (FAO 2020). Being an economical, rapid-growing, planktivorous-feeding habits, and a disease resistant fish, Nile tilapia became the most widely cultured fish (Ismail et al. 2021). Egypt is a major Nile tilapia-producing country with a production capacity of 1048.276 tons in 2016, representing 80% of the total fish production (GAFRD 2018).

Nutrition represents nearly 60–70% of Nile tilapia aquaculture costs (Ismail et al. 2019). Therefore, enhancing feed quality and cost is an effective way of overcoming obstacles with Nile tilapia aquaculture. Water weeds, which have long been recognized as waste, are one of the cheapest and most abundant potential sources of protein. Presently, they could be used as an alternative feed to develop Nile tilapia-production system (Magouz et al. 2020).

Fermentation strategy was followed to handle the environmental impact of agricultural wastes and provide safe and affordable feedstuff. According to Zhang et al. (2017) and Sun et al. (2015), fermentation techniques improved vitamin availability, protein, and fiber quality, besides, palatability of feedstuff. Moreover, it improved the innate immunity of fish (Siddik et al. 2019). Bacterial fermentation using useful bacteria such as *Bacillus subtilis* was revealed to have antitumor and immunomodulatory effects. Interestingly, it enhances the growth and viability of intestinal lactic acid bacteria (Liu et al. 2012).

Probiotic strategies are a promising alternative to antibiotics, either as growth promoters or modulators for fish immunity, so this study may pave the way to the possibility of using *Bacillus subtilis*-fermented *Azolla* in Nile tilapia feed through assessing its influence on fish growth performance, hemato-immunological scheme, intestinal digestive enzymes, disease resistance, and intestinal histomorphometry, thereby detecting the ideal dietary inclusion level.

## Material and methods

### Fermented Azolla pinnata preparation

For inoculum preparation, cultured *B. subtilis* E20 was received from the Microbiological Resources Center, Faculty of Agriculture, Ain Shams University, Cairo, Egypt, and enriched by immersion in nutrient broth for 24 h at 40 °C following the instruction by Juan and Chou (2010). Then cells were harvested by centrifugation (3000 ×*g* for 20 min) as reported by Sun et al. (2011). CFU was determined using spread plate method (Al-Harbi and Uddin 2004). Fermentation of *A. pinnata* performed as prescribed by Cheng et al. (2017) with minor modifications. Briefly, after inoculum preparation, each 1 kg *A. pinnata* was autoclaved at 121 °C for 20 min and inoculated with 250 ml *B. subtilis* inoculum. Then, inoculated *A. pinnata* incubated at 40 °C for 72 h. Finally, the fermented feed was spread on a foil sheet in a hot air oven and kept at 40 °C until the moisture content decreased to obtain two constant weights. Fermented *A. pinnata* was analyzed chemically to demonstrate its crude protein, ether extract, fiber, ash, and amino acid contents as shown in Tables 1 and 2.

**Table 1:** Ration formulation of experimental diets

Ingredient (%)	BSFA (%)				
	0	15	30	45	60
FM^1^	9	9	9	9	9
SBM^2^	22	18	18	16	13
CGM^3^	14	14	10.5	9	8
wheat bran	14.32	10.42	8	4.8	1
BSFA^4^	0	15	30	45	60
Maize	15.5	11	8.5	4.86	2
rice polish	16.55	13.05	8.13	5	1.35
α-cellulose	4.68	4.68	3.12	1.7	0.4
Oil	1.5	2.5	2.5	2.5	3.1
Mineral^*^	0.1	0.1	0.1	0.1	0.1
Vitamins^**^	0.1	0.1	0.1	0.1	0.1
Vitamin C	0.1	0.1	0.1	0.1	0.1
NaCl	0.05	0.05	0.05	0.05	0.05
Antimycotoxin	0.1	0.1	0.1	0.1	0.1
CMC^5^	0.5	0.5	0.5	0.5	0.5
Dicalcium P	1	1	1	1	1
DL-Methionine	0.3	0.1	0.1	0.04	0.1
Lysine-HCl	0.2	0.2	0.2	0.15	0.1
L-Threonine	0	0.1	0	0	0

^1^Moroccan fish meal 63% CP, ^2^soybean meal 48% CP, ^3^corn gluten meal 60%CP, ^4^*Bacillus subtilis* fermented Azolla 22.3% CP, ^5^xarboxy methyl cellulose. ^*^Minerals (Egypt pharma company) each 1.25 kg of this product provide the following: 5000 mg copper; 5 mg cobalt; 5000 mg iodine; 100 mg selenium; 30,000 mg iron; 40,000 mg magnesium; 10,000 mg manganese; 150,000 mg zinc; calcium carbonate ad to 1000 gm. ^**^Vitamins (Egypt pharma company) each 1.25 kg of this product provide the following: 5,000,000 IU Vit. A; 1,000,000 IU Vit. D3; 50,000 mg Vit. E; 10,000 mg K3; 20,000 mg B1; 20,000 mg B2; 20,000 mg B6; 20 mg B12; 100,000 mg niacin; 5000 mg folic; 100 mg biotin; 50,000 mg pantothenic acid; calcium carbonate ad to1000 gm

**Table 2:** Chemical analysis of experimental diets

Items	AM^*^	BSFA^**^	BSFA (%)				
			0	15	30	45	60
CP%	21.5	22.3	30.18	30.22	30.17	30.46	30.47
EE%	3.8	3.5	6.45	6.95	6.37	6.04	6.19
Ash%	15.9	16.5	5.19	6.85	8.73	10.59	12.31
NDF%	35.55	31.43	8.93	9.79	10.03	10.37	10.87
Gross energy (kJ kg^−1^)^1^	1405.62	1427.59	1816.73	1788.65	1765.17	1768.63	1752.62
Amino acids (g 100g^−1^ DM)							
Arginine	1.13	1.53	1.67	1.57	1.75	1.86	1.89
Histidine	0.45	0.43	0.74	0.72	0.71	0.71	0.69
Isoleucine	0.93	0.95	1.30	1.29	1.32	1.34	1.34
Leucine	1.55	1.60	2.94	2.29	2.74	2.72	2.66
Methionine	0.44	0.56	0.96	0.80	0.79	0.76	0.85
Lysine	0.99	0.96	1.40	1.40	1.47	1.48	1.47
Phenylalanine	1.01	0.98	1.13	1.19	1.15	1.20	1.23
Threonine	0.88	0.93	1.12	1.23	1.17	1.21	1.23
Valine	1.19	1.27	1.51	1.55	1.56	1.61	1.63

^1^Gross energy calculated using gross calorific values of 23.63, 39.52, and 17.15 kJ g^−1^ for protein, fat, and carbohydrate, respectively as recorded by (Brett, 1973). ^*^Azolla meal. ^**^Bacillus subtilis-fermented *Azolla*

### Experimental diets and protocol

Five isonitrogenous (30% crude protein) and isocaloric (17.78 Kj g^−1^) gross energy practical diets were prepared. The first one was the control diet, free of *B. subtilis*-fermented *Azolla* (BSFA0), while the other four diets were formulated to contain graded levels of BSFA of (15 to 60%) in terms of BSFA15, BSFA30, BSFA45, and BSFA60. Before the beginning of the experiment, tested ingredients and final diets were analyzed to demonstrate their chemical composition as presented in Table 2.

### Fish husbandry and feeding trial

The experiment was conducted in 15 glass aquaria (70 × 40 × 50cm) under five treatments (*n* = *3*). Twenty Nile tilapia fish with initial weight (15.60 ± 0.17 g) were stocked in each aquarium. Before the start of the experiment, fish were kept in aquaria for 2 weeks for accommodation. To maintain water quality, aquaria were provided with pumps for continuous aeration; additionally, water was changed daily at 40% rate. Moreover, water parameters including temperature, level of ammonia, dissolved oxygen, nitrite, nitrates, and pH were monitored regularly and maintained at (27 °C ± 1.3), (0.06 ± 0.01 mg L^−1^), (> 6.0 mg L^−1^), (0.04 ± 0.01 mg L^−1^), (15.8 ± 1.8 mg L^−1^), and (6.4 ± 0.5) throughout the experiment, respectively. Fish were fed diets up to satiation twice daily at 8:00 and 13:00 h for 95 day. During the experiment, fish were weighed every 2 weeks to assess fish growth performance throughout the process.

### Sampling

Before the final sampling, the fish were starved for 24 h, weighed individually, and then anesthetized using 50 mg l^−1^ 4-allyl methoxy phenol. Then, blood samples were obtained from the caudal veins of three fish per aquarium (9 fish/group) using sterile syringes and were divided into two halves. The first one was kept in a heparinized microtube for hematological and phagocytosis analyses, while the other half was transferred to nonheparinized microtube, and centrifugated at 3000 rpm for 15 min to obtain serum, then kept at −20 °C until use for biochemical analysis. Then, these nine fish sampled were preserved at −20 °C for proximate chemical analysis. The viscera and liver of three fish/group were removed and weighted to calculate the hepato-somatic index (HSI) and the viscero-somatic index (VSI). Three fish per replicate (9 fish/group) were sampled, and the intestines were dissected and rinsed with phosphate-buffered saline (PBS) (pH 7.5; 1 g per10 ml), homogenized and centrifuged at 8000 rpm for 5 min. Next, supernatant was collected and maintained at liquid nitrogen until the analysis of digestive enzyme activity. For histomorphometric evaluation, 2 fish per aquarium were eviscerated and tissue specimens obtained from the anterior, middle, and terminal parts of the intestine were preserved in Bouin’s solution.

### Growth and somatic indices

The following variables were calculated:

$$\begin{array}{l}\mathrm{Specific}\;\mathrm{growth}\;\mathrm{rate}\;\left(\mathrm{SGR}\%;\mathrm{day}^{-1}\right)=\left\{\left[\log\;\mathrm e\;\left(\mathrm{final}\;\mathrm{weight}\right)-\log\;\mathrm e\;\left(\mathrm{initial}\;\mathrm{weight}\right)\right]\right./\\\mathrm{duration}\;\left(95\;\mathrm{days}\right)\times100\;\left(\log\;\mathrm e=\mathrm{natural}\;\mathrm{logarithm}\;\mathrm{reading}\right)\end{array}$$

$$\mathrm{Weight}\;\mathrm{gain}\;(\%)=\left(\mathrm{final}\;\mathrm{weight}-\mathrm{initial}\;\mathrm{weight}\right)\times100/\mathrm{initial}\;\mathrm{weight}$$

$$\mathrm{Survival}\;(\%)=\left(\mathrm{final}\;\mathrm{no}.\;\mathrm{of}\;\mathrm{fish}/\mathrm{initial}\;\mathrm{no}.\;\mathrm{of}\;\mathrm{fish}\right)\times100$$

$$\mathrm{Feed}\;\mathrm{intake}\left(\mathrm{FI};\;\mathrm g\;\mathrm{fish}^{-1}\;95\mathrm{days}^{-1}\right)=\left(\mathrm{dry}\;\mathrm{diet}\;\mathrm{given}-\mathrm{dry}\;\mathrm{remaining}\;\mathrm{diet}\;\mathrm{recovered}\right)/\mathrm{no}.\;\mathrm{of}\;\mathrm{fish}$$

$$\mathrm{Protein}\;\mathrm{efficiency}\;\mathrm{ratio}\;(\mathrm{PER})=\mathrm{live}\;\mathrm{weight}\;\mathrm{gain}\;(\mathrm g)/\mathrm{dry}\;\mathrm{protein}\;\mathrm{intake}\;(\mathrm g)$$

$$\mathrm{Feed}\;\mathrm{efficiency}\;\mathrm{ratio}\;(\mathrm{FER})=\mathrm{live}\;\mathrm{weight}\;\mathrm{gain}\;(\mathrm g)/\mathrm{dry}\;\mathrm{feed}\;\mathrm{intake}\;(\mathrm g)$$

$$\mathrm{VSI}=\left(\mathrm{viscera}/\mathrm{body}\;\mathrm{weight}\right)/100$$

$$\mathrm{HSI}=\left(\mathrm{liver}/\mathrm{body}\;\mathrm{weight}\right)/100$$

### Hemtological and serum biochemical indices

Red blood cells (RBCs), hemoglobin (Hb), hematocrit (Hct), and white blood cells (WBCs) were determined using an automatic blood cell counter (Exigo-Vet., Boule Medical AB Inc., Stockholm, Sweden).

The total serum protein was determined as previously described by Doumas et al. (1981). Serum aspartate aminotransferase (AST), alanine aminotransferase (ALT), and creatinine were determined by a microlab 300 chemistry analyzer, on the basis of the kit’s manufacturer instructions (Spinreact Co. Spain).

Serum lysozyme activity was evaluated, as reported by Ellis (1990), with minor modifications. Briefly, lyophilized *Micrococcus lysodeikticus* (0.03%) in 0.05 mM arrangement phosphate buffer (pH 6.2) was used as the substrate. Using lyophilized hen egg white lysozyme (Sigma), a standard curve was drawn and the rate of change in turbidity was measured at 30- and 270-s interval at 530 nm using a Bio-Rad Microplate Reader.

Phagocytosis assay will be calculated as assayed by Jensch-Junior et al. (2006) from the following equations:

$$\mathrm{Phagocytic}\;\mathrm{activity}=\mathrm{macrophages}\;\mathrm{that}\;\mathrm{contain}\;\mathrm{yeast}/\mathrm{total}\;\mathrm{number}\;\mathrm{macrophages}\times100$$

$$\mathrm{Phagocytic}\;\mathrm{index}=\mathrm{number}\;\mathrm{of}\;\mathrm{yeast}\;\mathrm{cells}\;\mathrm{phagocytized}/\mathrm{macrophage}\;\mathrm{cells}\;\mathrm{phagocytizing}\;\mathrm{counted}.$$

Superoxide dismutase (SOD), glutathione peroxidase (GPx), and malondialdehyde (MDA) levels in serum were determined using commercial diagnostic kits (Cusabio Biotech Co., Ltd; China), following the manufacturer’s instructions.

### Proximate chemical analysis

Proximate chemical analysis for the ingredients, diets, and fish was carried out as prescribed by the Association of Official Analytical Chemists methods (AOAC 2010). The moisture content was determined by drying the samples in a hot-air oven at 60 °C for 48 h, and the ash content was determined by incinerating the samples in muffle furnace (Thermolyne Corporation, Dubuque, Iowa, USA) at 550 °C for 6 h. After acid digestion, crude protein content (*N* × 6.25) was investigated using the Kjeldahl method (Kjeltec™ 8400 fully automated analyzer). Fat and fiber were determined using the ANKOM technology method.

### Digestive enzymes activity investigation

According to Lowry et al. (1951), the total protein content was evaluated using bovine serum albumin (BSA) as a standard. The protease activity was determined using Folin-Ciocalteu phenol reagent, while the amylase activity was measured using iodine solution to detect nonhydrolyzed starch as prescribed by Worthington (1988). In addition, the lipase activity was determined according to the protocol assayed by Jin (1995), using olive oil as a standard. The protease and amylase activities were expressed as “units per mg of protein,” while the activity of lipase was expressed as “units per gram of intestinal content.”

### Histomorphometric examination

The collected samples for histological observation were immediately fixed in Bouin’s solution and then (after 18 h) dehydrated through ascending grades of an alcohol solution series, cleared in xylene, embedded in paraffin, cut into several 5-µm-thick sections using a rotary microtome (RM 20352035; Leica Microsystems, Wetzlar, Germany), and mounted on coated slides. For general morphometry and goblet cell recognition, the paraffin sections were rehydrated and stained with hematoxylin and eosin (H&E) and periodic acid–Schiff (PAS), as previously described (Gewaily et al. 2021a). The stained sections were examined under a light microscope (Leica DM500; Leica Microsystems, Japan). The morphometric examination was performed using a computerized image analysis system (ImageJ; Bethesda, MD, USA) to examine the villus height, width, area, and the number of goblet cells. The data obtained were subjected to statistical analysis.

### Bacterial challenge

At the end of the experiment (95 days), 7 fish from each glass aquarium (21 fish/group) were injected I/P with the pathogenic *Aeromonas septicemia* (0.3 ml of 10^8^ cells/ml) according to Schaperclaus et al. (1992). To determine the competitive exclusion of pathogenic bacteria through the production of inhibitory compounds, the injected fishes were observed for 14 days to record the clinical picture and cumulative mortality percentage.

### Statistical analysis

Data were analyzed by one-way analysis of variance and recorded as the mean ± standard error of three replicates. *P* < 0.05 was considered statistically significant and multiple comparisons among means were made by Duncan’s new multiple range test using SPSS Statistics v. 24. Microsoft Excel 16 was used to assess the quadratic polynomial regression.

## Results

### Assessment of Azolla pinnata and Bacillus subtilis-fermented Azolla nutritional value

The nutritional value of *A. pinnata* and *BSFA* is summarized in Table 2. Interestingly, fermentation positively affected CP and fiber content of *A. pinnata*. CP% elevated efficiently from 21.5 to 22.3%, while NDF% diminished from 35.55 to 31.43. Moreover, gross energy of *BSFA* relatively improved by 1.54 % over nontreated *Azolla*. Furthermore, fermentation also improved arginine, methionine, threonine, and valine amino acid levels compared to crude *A. pinnata*. These values were considered while preparing our experimental diets to fulfill the nutrient requirement for Nile tilapia.

### Growth performance and somatic parameters evaluation

Regarding the growth indices, shown in Table 3, fermentation strategy promoted the growth profile including final body weight (FBW), body weight gain (BWG), feed conversion (FCR), and specific growth rate (SGR). Interestingly, BSFA incorporation at a 30% rate in tilapia feed revealed the highest FBW, BWG, and SGR (*P* < 0.05). Meanwhile, increased inclusion rate above 30% poses an obstacle against growth elaboration. BSFA60 group significantly recorded the lowest FBW, BWG, SGR, and PER values (*P* < 0.05). Curiously, BSFA30 gave the finest FCR records (*P* < 0.05). Meanwhile, compared to the control group, BSFA45 and BSFA60 groups exhibited higher and lower FCR values, respectively. In contrast, fermentation insignificantly influenced somatic indexes and survival percentage of the experimental fish (*P* > 0.05). Quadratic polynomial regression was applied to detect the optimal BSFA dietary level which produces the best FBW, BWG, SGR, PER, FCR, and FI records and was found to be 30.1%, 29.82%, 29.11%, 28.86%, 29.22%, and 29.92% respectively, as indicated in Table 8. The relationship between dietary BSFA level and maximum FBW, BWG, SGR, PER, FCR, and FI, shown in Table 8, was expressed by the regression equations as follows:

**Table 3:** Growth parameters, feed utilization and somatic indices of Nile tilapia fed *Bacillus subtilis* fermented *Azolla* for 95 days

Items	BSFA (%)				
	0	15	30	45	60
IW (g)	15.60 ± 0.15	15.50 ± 0.17	15.60 ± 0.17	16.03 ± 0.35	15.80 ± 0.21
FBW (g)	58.17 ± 0.91^c^	58.38 ± 0.72^c^	65.83 ± 0.35^a^	61.27 ± 0.39^b^	56.10 ± 0.36^d^
FI (g fish^−1^)	66.42 ± 0.62^c^	65.88 ± 0.59^c^	71.09 ± 0.63^a^	69.17 ± 0.63^b^	64.46 ± 0.31^d^
BWG (%)	272.82 ± 2.18^b^	276.71 ± 4.76^b^	322.06 ± 2.47^a^	282.49 ± 8.85^b^	255.13 ± 2.42^c^
FCR	1.56 ± 0.01^b^	1.54 ± 0.02^b^	1.42 ± 0.01^c^	1.53 ± 0.01^b^	1.59 ± 0.02^a^
SGR	1.38 ± 0.01^c^	1.39 ± 0.02^c^	1.52 ± 0.01^a^	1.44 ± 0.02^b^	1.33 ± 0.01^d^
PER	2.14 ± 0.02^bc^	2.17 ± 0.02^b^	2.35 ± 0.02^a^	2.18 ± 0.01^b^	2.08 ± 0.02^c^
Survival (%)	95.00 ± 2.89	96.10 ± 2.01	96.67 ± 1.67	96.67 ± 1.67	93.33 ± 1.67
HIS	1.43 ± 0.07	1.49 ± 0.16	1.38 ± 0.04	1.40 ± 0.04	1.53 ± 0.07
VSI	5.19 ± 0.07	5.49 ± 0.14	5.26 ± 0.24	5.15 ± 0.50	6.10 ± 0.26

^*^Values are means ± standard error (*n* = 3). Means with different small letter in the same column differ significantly *(P* < 0.05). *IW*, initial weight; *FBW*, final body weight; *BWG*, body weight gain; *ADG*, average daily gain; *FI*, feed intake; *FCR*, feed conversion ratio; *SGR*, specific growth rate; *PER*, protein efficiency ratio; *HSI*, hepatosomatic index; *VSI*, viscerosomatic index

$$\mathrm{FBW};\;\mathrm y=-0.0072\mathrm x^2+0.4254\mathrm x+56.947$$

$$\mathrm{BWG};\;\mathrm y=-0.0073\mathrm x^2+0.4208\mathrm x+41.41$$

$$\mathrm{SGR};\;\mathrm y=-0.0001\mathrm x^2+0.0082\mathrm x+1.3577$$

$$\mathrm{PER};\;\mathrm y=-0.0002\mathrm x^2+0.0109\mathrm x+2.1189$$

$$\mathrm{FCR};\;\mathrm y=0.0001\mathrm x^2-0.0071\mathrm x+1.5737$$

$$\mathrm{FI};\;\mathrm y=-0.0049\mathrm x^2+0.2905\mathrm x+65.32$$

### Hematology, immunity, and antioxidative status

As depicted in Table 4, hematological and serum biochemical assays including (Hb, Hematocrit, RBCs, WBCs, Total protein, ALT, AST, and creatinine) values did not indicate any significant changes or physiological abnormalities owing to dietary BSFA (*P* > 0.05).

**Table 4:** Hematological and serum biochemical parameters of Nile tilapia fed *Bacillus subtilis* fermented *Azolla* for 95 days

Items	BSFA (%)				
	0	15	30	45	60
Hb (g/dl)	5.98 ± 0.37	6.38 ± 0.32	6.20 ± 0.06	6.25 ± 0.02	6.10 ± 0.47
RBCs (10^6^/µl)	2.06 ± 0.08	2.26 ± 0.05	2.20 ± 0.13	2.11 ± 0.03	2.21 ± 0.22
Hematocrit (%)	30.03 ± 0.35	30.40 ± 0.60	30.08 ± 0.85	29.66 ± 0.28	29.81 ± 0.18
WBCs (10^3^/µl)	37.75 ± 0.66	37.07 ± 1.02	37.95 ± 0.50	37.59 ± 1.25	37.00 ± 0.39
Total protein (g/dl)	2.52 ± 0.01	2.59 ± 0.06	2.61 ± 0.10	2.57 ± 0.04	2.61 ± 0.06
ALT (U/L)	31.38 ± 0.32	31.53 ± 0.37	32.53 ± 0.65	31.16 ± 0.54	32.30 ± 0.65
AST (U/L)	188.57 ± 2.96	187.67 ± 2.19	190.67 ± 2.33	188.35 ± 1.86	186.33 ± 1.45
Creatinine (mg/dl)	0.25 ± 0.01	0.24 ± 0.02	0.23 ± 0.02	0.23 ± 0.01	0.24 ± 0.01

^*^Values are means ± standard error (*n* = 3). Means with different small letter in the same column differ significantly (*P* < 0.05). *Hb*, hemoglobin; *RBCs*, red blood cells; *WBCs*; white blood cells; *GPT*, glutamic pyruvic transaminase; *GOT*, glutamic oxaloacetic transaminase

Curiously, BSFA was assayed to influence fish immunity status in terms of phagocytosis and lysozyme activities as exhibited in Table 5. Interestingly, level of dietary BSFA gradually increased the lysozyme activity until it reaches its peak at BSFA30 group (*P* < 0.05). Additionally, tilapia fed with BSFA30 recorded the highest phagocytic activity, followed by these feds with BSFA45. Meanwhile, the BSFA15 group expressed the same phagocytic activity values compared to the control group (*P* > 0.05). In contrast, BSFA60 showed the lowest phagocytic and lysozyme activity values. On regard with phagocytic index, it greatly promoted by increasing the dietary BSFA dietary level to 30% (*P* < 0.05), then, began declining after that. BSFA15 and BSFA60 showed insignificant improvement in relation to the control group (*P* > 0.05).

**Table 5:** Immunological response and oxidative status of Nile tilapia fed *Bacillus subtilis* fermented *Azolla* for 95 days

Items	BSFA (%)				
	0	15	30	45	60
Lysozyme activity (U/ml)	136.14 ± 4.02^cd^	156.47 ± 4.65^ab^	170.03 ± 5.55^a^	142.13 ± 4.59^bc^	125.40 ± 5.14^d^
Phagocytic activity (%)	45.80 ± 0.29^c^	46.99 ± 0.36^c^	59.82 ± 0.55^a^	51.58 ± 0.71^b^	40.40 ± 0.48^d^
Phagocytic index	2.33 ± 0.08^c^	2.33 ± 0.07^c^	2.70 ± 0.04^a^	2.50 ± 0.03^b^	2.18 ± 0.03^c^
SOD (U/ml)	4.85 ± 0.13^b^	4.87 ± 0.27^b^	4.84 ± 0.24^b^	5.08 ± 0.12^ab^	5.27 ± 0.19^a^
GPx (mU/mL)	0.24 ± 0.01^b^	0.21 ± 0.02^b^	0.25 ± 0.04^b^	0.26 ± 0.01^b^	0.32 ± 0.12^a^
MDA (mmol/mL)	10.80 ± 1.18	10.44 ± 0.43	10.36 ± 0.56	10.36 ± 0.64	10.50 ± 1.35

^*^Values are means ± standard error (*n* = 3). Means with different small letter in the same column differ significantly (*P* < 0.05)

The antioxidative response of Nile tilapia fed on graded levels of BSFA is presented in Table 5. The serum MDA activity was not influenced by dietary doses of BSFA (*P* > 0.05). On contrary, BSFA inclusion level increase to a 60% rate, significantly promoted SOD and GPx levels in serum compared to the other groups (*P* < 0.05). Following the quadratic polynomial regression, presented in Table 8, the best dietary BSFA level which gives rise to the optimal lysozyme activity, phagocytic activity, phagocytic index*, SOD, and GPx* levels is recorded to be 29.95%, 29.01%, 29.12%, 12.03, and 18.55, respectively, and expressed by the regression equations as follows:

$$\mathrm{Lysozyme}\;\mathrm{activity};\;\mathrm y=-0.0367\mathrm x^2+2.1539\mathrm x+130.95$$

$$\mathrm{Phagocytic}\;\mathrm{activity};\;\mathrm y=-0.0145\mathrm x^2+0.8312\mathrm x+43.616$$

$$\mathrm{Phagocytic}\;\mathrm{index};\;\mathrm y=-0.0004\mathrm x^2+.0222\mathrm x+2.2611$$

$$\mathrm{SOD};\;\mathrm y=0.0002\mathrm x^2-0.0046\mathrm x+4.8591$$

$$\mathrm{GPx};\mathrm{ y}=5\mathrm{E}-05{\mathrm{x}}^{2}-0.002\mathrm{x}+0.2443$$

### Carcass composition and quality

Data shown in Table 6 proved that body chemical composition (CP%, EE%, NDF%, and ash%) of the tested tilapia was insignificantly affected by dietary BSFA level (*P* < 0.05).

**Table 6:** Proximate chemical analysis of Nile tilapia fed *Bacillus subtilis* fermented *Azolla* for 95 days

Items	AM^*^	BSFA^**^	BSFA (%)				
			0	15	30	45	60
CP%	21.5	22.3	30.18	30.22	30.17	30.46	30.47
EE%	3.8	3.5	6.45	6.95	6.37	6.04	6.19
Ash%	15.9	16.5	5.19	6.85	8.73	10.59	12.31
NDF%	35.55	31.43	8.93	9.79	10.03	10.37	10.87
Gross energy (kJ kg^−1^)^1^	1405.62	1427.59	1816.73	1788.65	1765.17	1768.63	1752.62
Amino acids (g 100g^−1^ DM)							
Arginine	1.13	1.53	1.67	1.57	1.75	1.86	1.89
Histidine	0.45	0.43	0.74	0.72	0.71	0.71	0.69
Isoleucine	0.93	0.95	1.30	1.29	1.32	1.34	1.34
Leucine	1.55	1.60	2.94	2.29	2.74	2.72	2.66
Methionine	0.44	0.56	0.96	0.80	0.79	0.76	0.85
Lysine	0.99	0.96	1.40	1.40	1.47	1.48	1.47
Phenylalanine	1.01	0.98	1.13	1.19	1.15	1.20	1.23
Threonine	0.88	0.93	1.12	1.23	1.17	1.21	1.23
Valine	1.19	1.27	1.51	1.55	1.56	1.61	1.63

^1^Gross energy calculated using gross calorific values of 23.63, 39.52, and 17.15 kJ g^−1^ for protein, fat and carbohydrate, respectively as recorded by (Brett, 1973). ^*^Azolla meal. ^**^Bacillus subtilis-fermented *Azolla*

### Intestinal digestive enzyme assay

The intestinal digestive enzyme activities are presented in Table 7. Supplementation of BSFA at a 30% rate greatly improved the amylase activity (*P* < 0.05). Meanwhile, the BSFA15, BSFA45, and BSFA60 groups showed lower amylase activity values than the control group. Ironically, Nile tilapia fed on BSFA15 diet exhibited the highest protease activity level (*P* < 0.05), followed by those fed on the control diet and BSFA30. However, fermentation strategy did not significantly affect the lipase activity (*P* > 0.05). As recorded in Table 8, the optimal level of *BSFA* to provide the best amylase and protease activity is 25.66% and 22.85%, where regression equations were as follows:

**Table 7:** Intestinal digestive enzymes activities of Nile tilapia fed *Bacillus subtilis* fermented *Azolla* for 95 days

Items	BSFA (%)				
	0	15	30	45	60
Lipase activity (U/g intestinal content)	10.20 ± 0.61	9.61 ± 0.45	9.27 ± 0.66	9.56 ± 1.31	9.47 ± 0.56
Amylase activity (U/mg protein)	8.10 ± 0.23^b^	7.80 ± 0.37^bc^	10.11 ± 0.51^a^	7.96 ± 0.37^bc^	6.76 ± 0.33^c^
Protease activity (U/mg protein)	9.20 ± 0.60^ab^	10.28 ± 0.79^a^	9.27 ± 0.66^ab^	9.56 ± 0.43^ab^	8.14 ± 0.25^b^

^*^Values are means ± standard error (*n* = 3). Means with different small letter in the same column differ significantly (*P* < 0.05)

**Table 8:** Regression analysis based on different parameters of Nile tilapia fed *Bacillus subtilis* fermented *Azolla* for 95 days*

Item	Regression equation	*R*^2^	Optimal dose (%)
FCR	*y* = 0.0001*x*2 − 0.0071*x* + 1.5737	*R*^2^ = 0.6663	29.22
FI (g fish^−1^)	*y* = −0.0049*x*^2^ + 0.2905*x* + 65.32	*R*^2^ = 0.5978	29.92
BWG (g)	*y* = −0.0073*x*^2^ + 0.4208*x* + 41.41	*R*^2^ = 0.6619	29.82
FBW (g)	*y* = −0.0072*x*^2^ + 0.4254*x* + 56.947	*R*^2^ = 0.6551	30.10
SGR	*y* = −0.0001*x*^2^ + 0.0082*x* + 1.3577	*R*^2^ = 0.7115	29.11
PER	*y* = −0.0002*x*^2^ + 0.0109*x* + 2.1189	*R*^2^ = 0.6858	28.86
Lysozyme activity (U/ml)	*y* = −0.0367*x*2 + 2.1539*x* + 130.95	*R*^2^ = 0.784	29.95
Phagocytic activity (%)	*y* = −0.0145*x*^2^ + 0.8312*x* + 43.616	*R*^2^ = 0.7255	29.01
Phagocytic index	*y* = −0.0004*x*^2^ + 0.0222*x* + 2.2611	*R*^2^ = 0.6731	29.12
SOD (U/ml)	*y* = 0.0002*x*^2^ − 0.0046*x* + 4.8591	*R*^2^ = 0.959	12.03
GPx (mU/mL)	*y* = 5E-05*x*^2^ − 0.002*x* + 0.2443	*R*^2^ = 0.9035	18.55
Amylase activity (U/mg protein	*y* = −0.002*x*^2^ + 0.1021*x* + 7.7594	*R*^2^ = 0.5772	25.66
Protease activity (U/mg protein)	*y* = −0.0012*x*^2^ + 0.0515*x* + 9.3312	*R*^2^ = 0.7509	22.85

^*^The parameters showed significant differences (*P* < 0.05) are selected to be represented in the table

$$\mathrm{Amylase}\;\mathrm{activity};\;\mathrm y=-0.002\mathrm x^2+0.1021\mathrm x+7.7594$$

$$\mathrm{Protease}\;\mathrm{activity};\;\mathrm y=-0.0012\mathrm x^2+0.0515\mathrm x+9.3312$$

### Histomorphometry

**Fig. 1:**
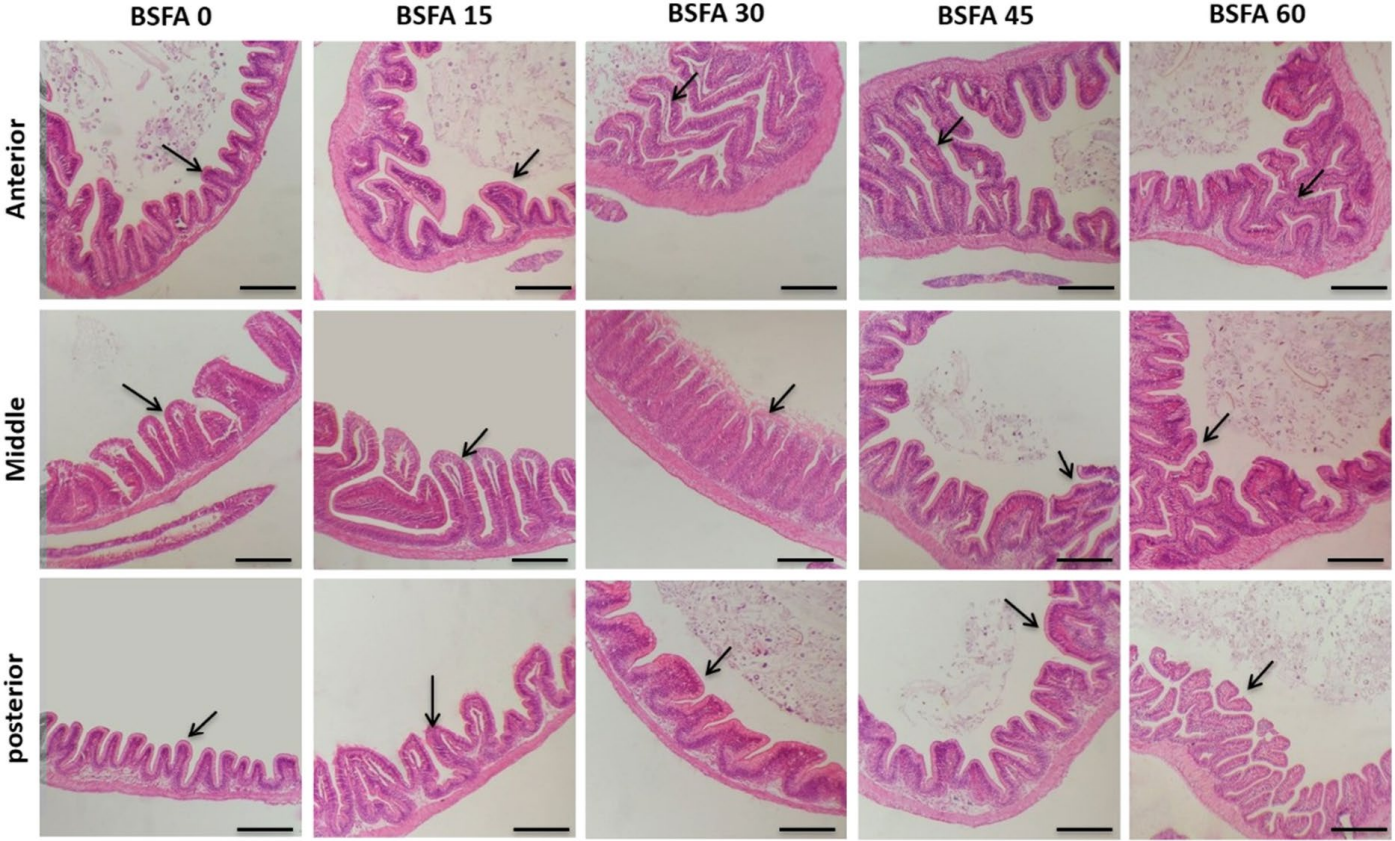
Histomicrograph of Nile tilapia intestine through the anterior, middle, and posterior segments in the control non-treated group as well as BSFA-fed groups (15%, 30%, 45%, and 60% respectively). The histological structures displayed regular arrangement of the layers of intestinal wall and intestinal villi (arrow). There was a noteworthy upgrading in the villus height, villus width, and villus area with increased level of BSFA. Stain H&E. Bar = 200 µm

**Fig. 2:**
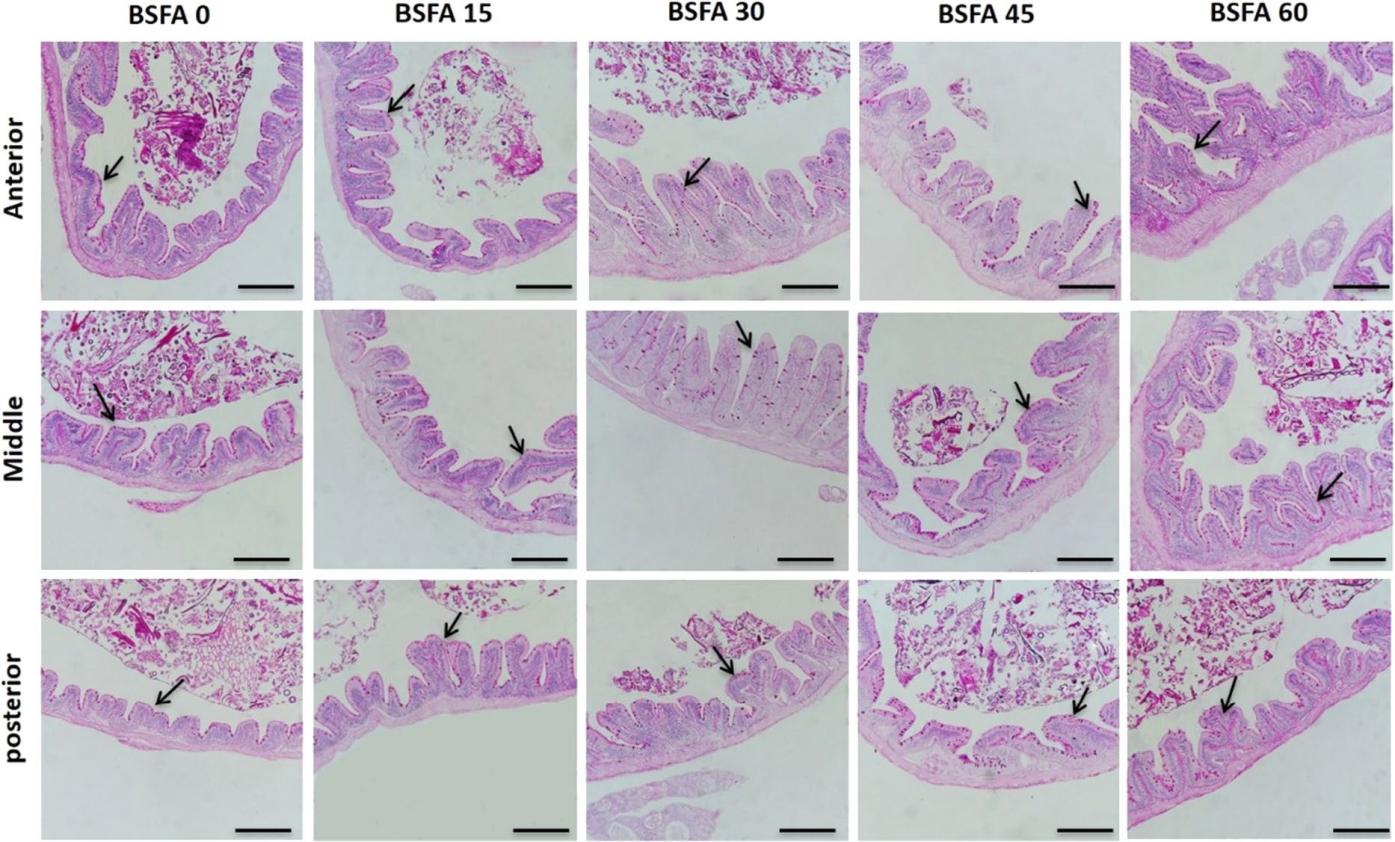
Histomicrograph of Nile tilapia intestine through the anterior, middle, and posterior segments in the control non-treated group as well as BSFA-fed groups (15%, 30%, 45%, and 60% respectively). The histological structures displayed normal arrangement of PAS-positive goblet cells. The number of goblet cells (arrow) increased all over the entire length of the intestine with increased level of BSFA. Stain PAS. Bar = 200 µm

**Fig. 3:**
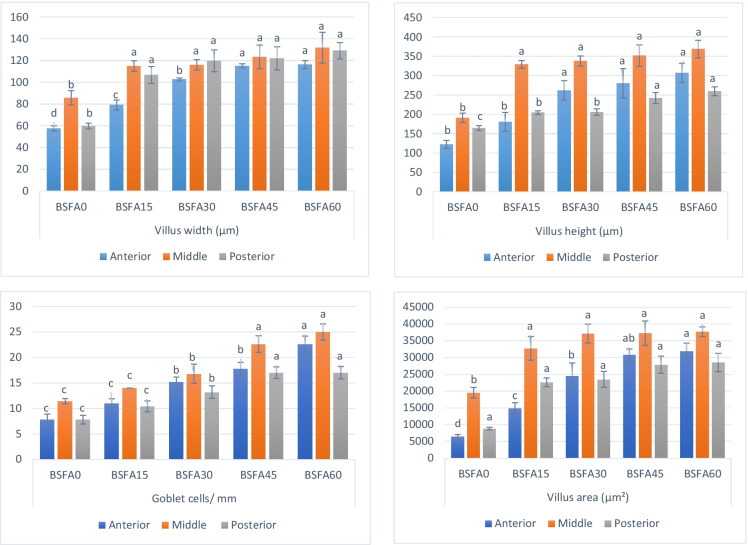
Effect of dietary *Bacillus subtilis* fermented Azolla (BSFA) on villus width, villus height, number of goblet cells and villus area of Nile tilapia intestine. Values are means ± standard error (*n* = 5). Means with different small letter on columns differ significantly (*P* < 0.05)

Histological assessment of the intestine supported the enhancement effect of BSFA. The morphological study of all experimental groups showed a normal structure of a four-layered-intestinal wall (mucosa, submucosa, muscularis, and serosa) (Figs. 1 and 2). The intestinal villi and associated crypts appeared healthy without any inflammatory or deteriorating changes. PAS staining revealed that the goblet cells that were appropriately arranged (Fig. 2). The histomorphometry assay of the intestines revealed a significant upgrade (*P* < 0.05) in all investigated parameters (the villus height, width, and area) in the BSFA-treated groups in association with increased levels of BSFA (Fig. 3). Moreover, there was obvious branching of the intestinal mucosa with a gradual increase in the goblet cell number.

### Disease resistance

**Fig. 4:**
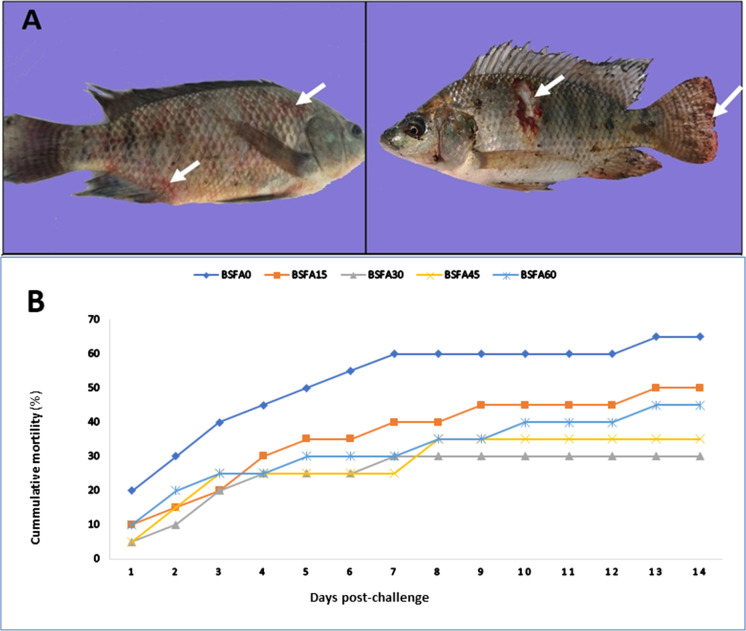
**A** Cutaneous ulcers appear on Nile tilapia body after challenging with *A. septicemia* (arrow); hemorrhagic patches (arrow) on different parts of the body; **B** cumulative mortality (%) of Nile tilapia fed graded level of dietary *Bacillus subtilis* fermented Azolla for 95 days and then challenged with *A. septicemia* for 14 days

The clinical findings showed skin darkening, focal hemorrhages at the base of fins, diffuse hemorrhage under the skin, ulcerative degradation and skin depigmentation, inflamed vent, swollen abdomen, and fin rot (Fig. 4A). BSFA-fed Nile tilapia had a low mortality rate compared to the control. The cumulative mortality curve (Fig. 4B) indicated that dietary BSFA (15–60%) was protective against *Aeromonas septicemia* infection in Nile tilapia. Oppositely experimental Nile tilapia fed on BSFA30-Nile tilapia showed nearly half mortalities (30%) observed in the control group (65%).

## Discussion

The overpricing of aquafeed materials coincided with their severe shortage, prompted scientists to look for less expensive, readily available, and nontraditional feedstuff that can yield heathy fish with high productivity. *A. pinnata* is one of the most important macrophytes which provide appropriate growth levels, with a substantial protein content (20–30%; dry weight) (Abou et al. 2007). Several studies were conducted to assess the nutritional value of *A. pinnata* as a replacer for several dietary components (Fasakin and Balogun 1998; Chareontesprasit and Jiwyam 2001; Gangadhar et al. 2017; and Magouz et al. 2020). As a nutritive and productive microphyte, *A. pinnata* was not practically examined as a fermented aquafeed. Our unique study was demonstrated to monitor the nutritive value of *A. pinnata* and assessed its influence on intestinal digestive enzymes and morphometry in Nile tilapia, as well as its impact on immune response and antioxidative status in vivo. Furthermore, our results stated that up to 30% of *BSFA* could be incorporated into Nile tilapia diets without deleterious effects on health, growth status and survival (%).

Interestingly, CP% elevated at a rate of 3.72%, and NDF% dropped at a rate of 11.59%. Moreover, amino acid profile of *BSFA* including arginine, methionine, threonine, and valine showed higher levels than raw *Azolla*. These results were also observed by Shiu et al. (2015), who documented that fermentation of soybean meal with *B. subtilis E20* (FSBM) enhanced its crude protein content and improved overall amino acids content compared to untreated one. In addition, *Bacillus*-fermented physic nut seed meal (*Jatropha curcas*) exhibited a significant increase in crude protein content, while the fiber content was diminished compared to nonfermented meal (Hassaan et al. 2017). This development in protein content may be attributed to the amino acid added during the fermentation process as a result of microbial protein synthesis (Belewu and Sam 2010; Ismail et al. 2021). Moreover, it was noted that protease enzymes released following fermentation shared in improving the amino acid profile (Hong et al. 2004; Lee et al. 2016). Additionally, in accordance with Hassaan et al. (2017), the diminishing in fiber content may be assigned to the fibrinolytic enzymes (cellulase, xylanase, amylases, hemicellulase, β-glycosidase, pectinases, and α-galactosidase) produced during the fermentation process.

Also, growth parameters including (FBW, BWG, FCR, SGR, and PER) revealed better values in fish fed BSFA at a rate of 30% than other experimental groups. Following Chareontesprasit and Jiwyam (2001), *A. pinnata* could be included in diets for Nile tilapia up to 15% without having detrimental effects on growth and feed utilization. Furthermore, dry *Azolla* was recommended at a level of 25% in *Tilapia zillii* diet without negative effects on growth and feed utilization (Abdel-Tawwab 2008). Interestingly, our progressive results could be explained by Liu et al. (2012) who reported that improving the growth of grouper fish (*Epinephelu scoioides*) fed *B. subtilis*-incorporated diet came back to the exoenzymes (proteases and lipases) secreted by *B. subtilis* which promotes nutrient digestibility, thereby improving growth. In addition, certain essential nutrients were produced by many *B. subtilis* spp. as amino acids and vitamins (Sanders et al. 2003; Rosovitz et al. 1998), which, in turn, improves growth and feed intake. In contrast, a higher inclusion level of BSFA beyond 30% poorly affected on growth. This could be attributed to the climbing in fiber content and some deleterious substances like phytates and phenolics which, in turn, influence digestibility and feed acceptability (Shamna et al. 2015).

Hematological parameters were stated to reflect fish’s general health condition and physiological status (Maita 2007). In the present study, BSFA did not show any specific trends in hematological (RBCs, Hb, WBCs, and hematoctit) and biochemical indices (AST, ALT, total protein, and creatinine). The same results were concluded in Japanese flounder (*Paralichthys olivaceus*) fed fermented soybean meal and squid by‐product blend (Abdul Kader et al. 2012). In parallel with our data, hematological and biochemical assays including Hb, PCV, total protein, AST, ALT, TG, and glucose showed insignificant changes in juvenile rainbow trout fed fermented protein concentrates (Moniruzzaman et al. 2018). These results proved that fermentation has a remarkable effect on the antinutritional factors (ANFs) which associated with iron and the amine group of amino acids and diminished their accessibility in host blood (Soltan et al. 2008).

Lysozyme is a potent bactericidal enzyme (Saurabh and Sahoo 2008). It has a destructive effect on gram-positive bacterial cell walls except opsonin which enhances a defense mechanism named phagocytosis. This mechanism is an efficient immunological response of the body against any infectious agent and can be assessed by determining the phagocytic activity and index (Harikrishnan et al. 2011). During this process, phagocytic cells produce reactive oxygen species (ROS) which are controlled by antioxidants to protect host cells. In this study, BSFA dietary inclusion dose showed a notable effect on nonspecific immune response and antioxidative status. Lysozyme activity, phagocytic activity, and phagocytic index reached their peaks in BSFA30 group. Similarly, fermented vegetable products improved lysozyme and phagocytic activities in Japanese flounders (Ashida and Okimasu 2005). In addition, oral administration of *B. subtilis* in gilthead seabream (*Sparus aurata*) for 2 weeks significantly increased its phagocytic activity (Dawood et al. 2016). On contrary, Nile tilapia fed *Azolla* meal did not show a significant effect on lysozyme activity, phagocytic activity, or phagocytic index (Magouz et al. 2020). SOD, GPx, and MDA are oxidative stress indicators which illustrate the oxidative status of the host. SOD considered the first antioxidative line of defense, preventing cells from damage by catalyzing H_2_O_2_ and removing the reactive oxygen species (ROS) (Wan et al. 2016). In addition, GPx helps sustaining host cells’ health via disproportionation of the toxic ROS to inactive oxygen molecules and hydrogen peroxide (Dawood et al. 2020). Conversely, MDA is a final product of lipid peroxidation and can be used as an indicator of oxidative damage (Ding et al. 2015). The present study revealed that BSFA had no observable effect on MDA, while SOD and GPx came to their climax in BSFA60 group. All these results suggest that BSFA modified the free oxygen radical scavenging capacity and developed the immune response of Nile tilapia. In line with our findings, juvenile black sea bream fed fermented soybean meal revealed a notable increase in *GPx* and SOD activity (Azarm and Lee 2014). Furthermore, white shrimp fed a *B. subtilis*-supplemented diet recorded higher SOD activity (Liu et al. 2014).

Regarding whole body chemical composition, the experimental tilapia BSFA-fed had no observable changes compared to the control group. Similarly, Moniruzzaman et al. (2018) stated that there were no significant changes in whole body chemical composition of juvenile rainbow trout fed fermented protein concentrates. Also, the same findings were noticed in *O. niloticus* fed graded levels of *A. pinnata* (Magouz et al. 2020). However, Abdel-Tawwab (2008) indicated that the gradual increase of *Azolla* meal levels in *Tilapia zillii*-tested diets showed declining in crude protein and lipid, with increasing moisture and ash contents.

As a widely accepted indicator of feed utilization and digestibility of the host, intestinal digestive enzyme activity was determined to assess the nutritive value and optimized level of BSFA for our experimental Nile tilapia (Ueberschär 1995). Curiously, amylase and protease enzymes are influenced by the level of BSFA included in diet and reached their summit in BSFA30 and BSFA15 groups, respectively, which agrees with the results found in the Nile tilapia group fed with a diet containing *Bacillus subtilis* spp. (Liu et al. 2017). As previously mentioned, wide range of *Bacillus* spp. was documented to produce exoenzymes which, in turn, participate in nutrients break down and digestion (Liu et al. 2009; Bandyopadhyay and Mohapatra 2009). On contrary, α-amylase, lipase, and trypsin activity in the intestine of juvenile black sea bream fish (*Acanthopagrus schlegeli*) were not influenced by dietary fermented soybean level (Azarm and Lee 2014). The activity of amylase and protease enzymes reduced with rising the levels of *A. pinnata*, which is compatible to findings of Magouz et al. (2020) in Nile tilapia. This can be assigned to the increase in oligosaccharides and nonstarch polysaccharides (NSPs), which, in turn, affect the viscosity and transit rate of digesta, and their binding action with bile salts leading to decreased bioavailability of nutrients and energy (Francis et al. 2001).

The histomorphometry in the current work supported the upgrading effect of BSFA, as previously mentioned. Intestinal morphometric analysis is a critical method for determining the impact of dietary supplements on the fish intestine’s absorption capacity, as well as local intestinal immunity, which reflects the fish’s overall immune condition (Abdel-Warith et al. 2021; Haygood and Jha 2018; Dawood et al. 2020). In addition, the incorporation of immune cells within the tissues of the fish gut plays a role in immunity (Gewaily et al. 2021b). However, the general morphology of all investigated groups showed a normal appearance, and the morphometric analysis clarified that the addition of BSFA to the Nile tilapia diet has a significant augmentation in the intestinal villi at the level of villus height, width, area, and goblet cell number. This may be due to the greater feed utilization in Nile tilapia BSFA-fed that was associated with increased intestinal villi surface area which is important for food absorption through the gut(Shukry et al. 2022; Zaki and Shatby 2015).

Disease resistance is the final indicator of host health status (Lim et al. 2009). A developed immune system can be proven by bacterial challenge test results. Our investigations revealed that dietary BSFA provided protection against *A. septicemia* infection in Nile tilapia. Similar results were reported in white leg shrimp (*Litopaeneus vannamei*) fed *B. subtilis*-fermented plant proteins and challenged with *Vibrio parahaemolyticus* (Hamidoghli et al. 2020). In addition, administration of *B. pumillus* improved the survival rate in Nile tilapia challenged with *A. hydrophyla* (Aly et al. 2008). The same findings were also obtained in rockfish (*Sebastes schlegeli*) challenged with *E. tarda* (Lee et al. 2016), rainbow trout (*Oncorhynchus mykiss*) challenged with *Aeromonas* spp. (Newaj-Fyzul et al. 2007), and cobia (*Rachycentron canadum*) challenged with *V. herveyi* (Geng et al. 2011). Thus, the present study showed that Nile tilapia fed with a diet incorporated with 30% BSFA for 95 days exhibited the lowest cumulative mortality (%) when challenged with *A. septicemia* for 14 days, followed by the BSFA45 group.

## Conclusion

We conclude that BSFA modifies Nile tilapia growth performance, innate immune response, and antioxidative status. Additionally, it upgrades feed utilization and nutrient digestibility coincided with improving intestinal enzymes and morphometry. Finally, it enhances Nile tilapia disease resistance against *A. septicemia.* The recommended dose of BSFA ranged from 29.01 to 30.10% according to the quadratic polynomial regression.

## Data Availability

The data that support the findings of this study are available from the corresponding author upon request.
